# Induction of Melanogenesis by Fosfomycin in B16F10 Cells Through the Upregulation of P-JNK and P-p38 Signaling Pathways

**DOI:** 10.3390/antibiotics9040172

**Published:** 2020-04-11

**Authors:** Sana Ullah, You Chul Chung, Chang-Gu Hyun

**Affiliations:** Department of Chemistry and Cosmetics, Jeju National University, Jeju 63243, Korea; sanaullah.su32@gmail.com (S.U.); jyc8385@hanmail.net (Y.C.C.)

**Keywords:** fosfomycin, melanogenesis, tyrosinase, JNK, p38, anti-whitening

## Abstract

Fosfomycin disodium salt (FDS), which is a water-soluble extract, is a bactericidal drug used to inhibit the synthesis of cells. Moreover, it has been found to be effective in the treatment of urinary tract infections. The present study was conducted to investigate the melanogenesis-stimulating effect of FDS in B16F10 cells. Several experiments were performed on B16F10 cells: the 3-(4,5-dimethylthiazol-2-yl)-2,5-diphenyl tetrazolium bromide (MTT) assay, the melanin content assay, the cellular tyrosinase activity assay, and Western blotting. FDS upregulated the activity of tyrosinase in a dose-dependent manner at a wide concentration range of 0–1 mg/mL, which showed no cytotoxicity. It also increased the melanin content and the activity of the microphthalmia-associated transcription factor (MITF), tyrosinase related protein 1 (TRP-1), and tyrosinase related protein 2 (TRP-2) enzymes in a dose-dependent manner. Western blotting results showed that FDS clearly upregulated the phosphorylation of c-Jun N-terminal kinases (JNK) and p38 pathways. These data are clear evidence of the melanogenesis-inducing effect of FDS in B16F10 murine melanoma cells.

## 1. Introduction

The skin is the largest organ of the body. In adults, it accounts for approximately 16% of the total body weight [1,2,3]. The primary function of the skin is to protect the internal organs from harmful chemicals, bacteria, ultra violet (UV) radiation, and many other harmful factors. In addition to this, it also protects many small biomolecules that play a vital role in metabolism in the body, such as DNA, RNA, and proteins. Therefore, any damage to the skin can cause many diseases in the body. People expose their body to sunlight to maintain adequate levels of vitamin D. However, depletion of the ozone layer is increasing due to increased air pollution, which, ultimately, increases the amount of harmful UV radiation that enters through the troposphere. UV radiation causes damages to humans, regardless of sex. Thus, it is essential to protect the skin from UV radiation [4,5,6,7]. Human skin is quite responsive to UV radiation, which generates many free radicals, reactive oxygen species, and other chain reactions that cause protein degradation, lipid peroxidation, and DNA oxidation [8].

Melanin is a black/brown coloring pigment present in almost every living organism from microorganisms to large mammals. The formation of this melanin pigment in the skin determines skin color, which plays a vital role in defense against harmful UV radiation and the free radicals inside the body [9,10,11,12]. Melanin is a heterogeneous biopolymer produced in specialized organelles (melanosomes) that not only produce but also store melanin. This production of melanin, known as melanogenesis, involves many enzymatic complex reactions [13,14,15]. Melanogenesis involves three main enzymes: tyrosinase, tyrosinase related protein 1 (TRP-1) and tyrosinase related protein 2 (TRP-2) [16]. In the melanogenesis reaction, tyrosinase acts as a very important enzyme, which catalyzes the oxidation of L-tyrosine as well as L-3,4-dihydroxyphenylalanine (L-DOPA) to DOPAquinone. DOPAquinone formed and underwent intramolecular cyclization to form leucoDOPAchrome, which is further oxidized to DOPAchrome [17,18,19,20,21]. In addition, tyrosinase converts DOPA chrome to 5,6-dihydroxyindole (DHI) and 5,6-dihydroxyindole-2-carboxylic acid (DHICA) [22,23]. Moreover, tyrosinase-related protein-1 (TRP-1) oxidizes 5,6-dihydroxyindole-2-carboxylic acid to indole-5,6-quinone-2-carboxylic acid in mice but has not been reported to have the same activity in humans. Tyrosinase-related protein-2 (TRP-2), which is also known as DOPAchrome tautomerase, is able to isomerize dopachrome to form DHICA [16]. These products are classified as eumelanin (brownish/black), which determine the color of the mammal [24,25,26,27,28]. The c-AMP-dependent protein kinase A (PKA) has been reported to activate microphthalmia-associated transcription factor (MITF) transcription via phosphorylation of cyclic adenosine monophosphate (cAMP) response element binding protein (CREB). MITF is a key factor in melanocyte development as it increases TRP-1 and TRP-2 production [26,29,30,31,32].

C-Jun N-terminal kinase (JNK), extracellular signal-regulated kinase (ERK), and p38 are the most important factors among the mitogen-activated protein kinases (MAPKs), with regard to melanin synthesis. In addition, according to recent studies, the phosphoinositide 3-kinase (PI3K)/protein kinase B (AKT) pathway is also related to melanogenesis. These are involved in MITF regulation [14,17,32,33,34,35].

Hair and skin color are considered significant signs of beauty among human beings. Different individuals prefer different colors of hair. This is possible due to an excess of melanin. Melanocytes release melanin. This takes place via an increase in the activity of tyrosinase and the expression of MITF, which simultaneously increase the content of melanin. The common use of anti-whitening dyes among humans has increased the interest, among researchers, in developing new anti-whitening hair dyes to increase melanin in the skin for its protection [36,37,38].

In the present study, the anti-hair whitening effects of different compounds were investigated, and fosfomycin disodium salt (FDS) was found to be the most effective. Previously, FDS was used as an antibiotic drug [39] as well as to promote absorption and secretion in urine, maintain blood levels [40], and enhance jejunal colonic absorption [41].

## 2. Results

### 2.1. Effect of FDS on Cell Viability

**Figure 1:**
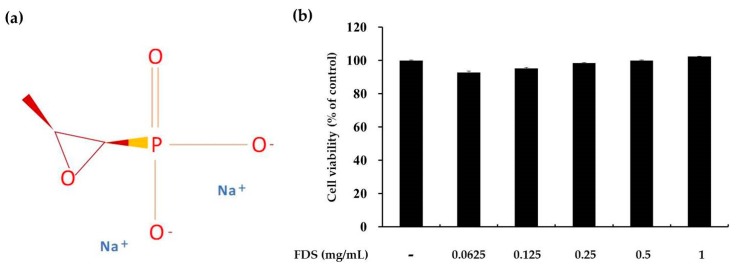
(**a**) Chemical structure of fosfomycin disodium salt (FDS). (**b**) The cell viability test that B16F10 cells were treated with FDS (0.0625, 0.125, 0.25, 0.5, and 1 mg/mL). Cell viability is expressed as percentages compared to the respective values obtained for untreated control cells. The data are presented as mean ± standard deviation (SD) of at least three independent experiments. SD: standard deviation.

The cytotoxicity of FDS at various concentrations ranging from 0.1 to 1 mg/mL was examined in B16F10 murine melanoma cells grown in 24-well plates and maintained in a humidified atmosphere. Cells (3 × 10^4^) treated with FDS and the 3-(4,5-dimethylthiazol-2-yl)-2,5-diphenyl tetrazolium bromide (MTT) solution were incubated at 37 °C and 5% CO_2_ for 2 h. MTT is a water-soluble salt that turns yellow when added to metabolically active cells as viable cells convert this dye into a water-insoluble dye called formazan. At the molecular level, this occurs due to reductive cleavage of the tetrazolium ring. Formazan is soluble in dimethyl sulfoxide (DMSO) and absorbs UV radiation at 450 ± 100 nm. The percentage of viable cells can be calculated from how much of the MTT was converted into formazan by the active cells. Greater formation of formazan indicates higher amount of active cells and vice versa. No significant differences on cell viability were observed with concentrations ranging from 0.0625 to 1 mg/mL (Figure 1). Treatment with FDS at concentrations from 1 to 2 mg/mL led to slight and dose-dependent increases in cell viability. Therefore, we used FDS at concentrations of 0.125, 0.25, and 0.5 mg/mL for further study.

### 2.2. Effect of FDS on Melanin Production

**Figure 2:**
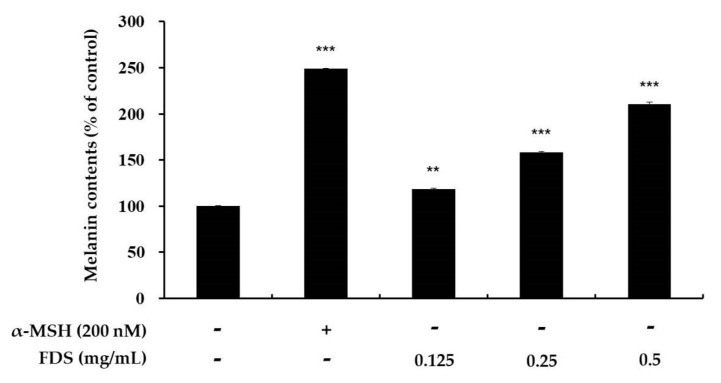
Effects of FDS on melanin content of B16F10 melanoma cells. The cells were treated with FDS (0.125, 0.25, and 0.5 mg/mL). α-melanocyte-stimulating hormones (α-MSH) (200 nM) was used as a positive control. The melanin content in the treated cells were expressed as percentages compared to the respective values obtained for the untreated control cells. The data are presented as mean ± standard deviation (SD) of at least three independent experiments. ** *p* < 0.01, *** *p* < 0.001 vs. untreated cell. SD: standard deviation.

To assess melanin content in cells treated with FDS, B16F10 murine melanoma cells at 1 × 10^5^ were seeded in 60-mm dishes, incubated for 24 h, and further incubated for 72 h with various concentrations of FDS (0.125–0.5 mg/mL). α-melanocyte-stimulating hormones (α-MSH) was used as a positive control. Melanin content was then measured. Melanin concentration percentage increased as FDS concentration increased. Previously, we found that FDS at this range of concentration showed no cellular toxicity. α-MSH-treated cells showed a marked increase in melanin content up to approximately 249% compared with that of the control, which was 100%. This indicated a more than 50% increase in melanin content. FDS also increased melanin content compared with the control group. In cells treated with FDS at 0.125 mg/mL, the melanin content slightly increased to 118%. Those treated with 0.25 mg/mL FDS had melanin content of 159%. Those treated with 0.5 mg/mL FDS had melanin content of almost 210% (Figure 2).

### 2.3. Effect of FDS on Cellular Tyrosinase Activity

**Figure 3:**
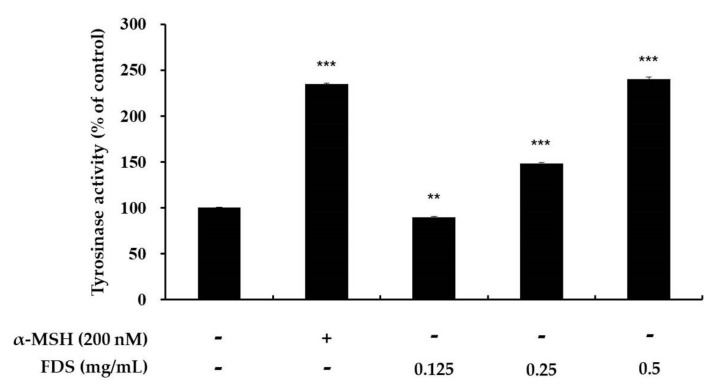
Tyrosinase activity in FDS-treated B16F10 melanoma cells. The cells were treated with various concentrations of FDS for 72 h, and α-MSH was used as a positive control. Data are presented as mean ± standard deviation (SD) of at least three independent experiments. ** *p* < 0.01, *** *p* < 0.001 vs. untreated cell. SD: standard deviation.

Tyrosinase plays a key role in the production of melanin, which is the skin-coloring pigment. Tyrosinase is involved in the first two steps of melanogenesis. Increasing the activity of tyrosinase will increase the production of melanin. For the assessment of cellular tyrosinase activity, B16F10 (1 × 10^5^) cells were seeded and treated with various concentrations of FDS (0.125–0.5 mg/mL). α-MSH was used as a positive control. Extracted proteins from these cells treated with FDS or α-MSH were then mixed with 15 mM L-DOPA. The results showed that the activity of tyrosinase increased in a dose-dependent manner. α-MSH increased the activity of tyrosinase up to 235% when compared with that in the control cells (100%), which contained no sample and no FDS. FDS at 0.125 mg/mL slightly decreased the activity of tyrosinase, when compared with the control. However, 0.25 mg/mL FDS increased the activity of tyrosinase up to 149% and 0.5 mg/mL FDS increased the activity up to 240%, when compared to the control (Figure 3).

### 2.4. Western Blotting Results

**Figure 4:**
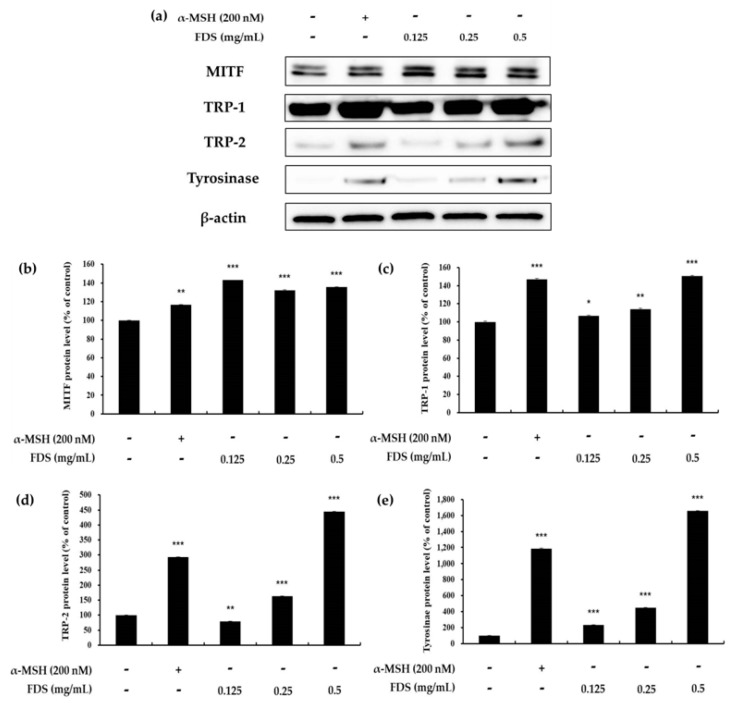
Effect of fosfomycin disodium salt (FDS) on microphthalmia-associated transcription factor (MITF), tyrosinase related protein 1 (TRP-1), tyrosinase related protein 2 (TRP-2), and tyrosinase expression in B16F10 cells. Cells were treated with various concentrations of FDS (0.125, 0.25, and 0.5 mg/mL). Protein levels were examined by Western blotting. (**a**) Result of Western blotting, and protein levels of (**b**) MITF, (**c**) TRP-1, (**d**) TRP-2, and (**e**) tyrosinase. Results are expressed as a percentage of the control. The data are presented as mean ± SD of at least three independent experiments. * *p* < 0.05, ** *p* < 0.01, *** *p* < 0.001 vs. untreated cell. SD: standard deviation. TRP: tyrosinase-related protein. MITF: microphthalmia-associated transcription factor.

To elucidate whether FDS enhances the expression of melanogenic proteins, we performed Western blot analysis. B16F10 (1 × 10^5^) cells were treated for 72 h with various concentrations of FDS (0.125 to 0.5 mg/mL). In the bicinchoninic acid (BCA) assay, 25 µg of proteins were separated by gel electrophoresis and then moved to a polyvinylidene fluoride (PVDF) membrane, which was then blocked with 5% skim milk and incubated with specific primary antibodies to detect the expression of each protein. Tyrosinase is a key enzyme in the melanogenesis pathway. The main function of tyrosinase is to convert L-tyrosine and L-DOPA into DOPAquinone. Therefore, the protein expression of this enzyme was assessed. As shown in Figure 4, the expression of the tyrosinase enzyme increased as the concentration of FDS increased. TRP-1 and TRP-2 play an important role as catalysts in promoting melanogenesis. The result also showed that the expression of these melanogenic enzymes increased by FDS in a dose-dependent manner. Furthermore, MITF is an enzyme well-known to play a key role in many pathways. It binds to the M-box within the tyrosinase promoter and increases tyrosinase expression. Upregulation of MITF ultimately induces melanogenesis. The result indicated that an FDS-treated cell increased the expression of MITF as compared to untreated cells (Figure 4).

### 2.5. Effect of FDS on AKT, JNK, and p38 Signaling Pathways

**Figure 5:**
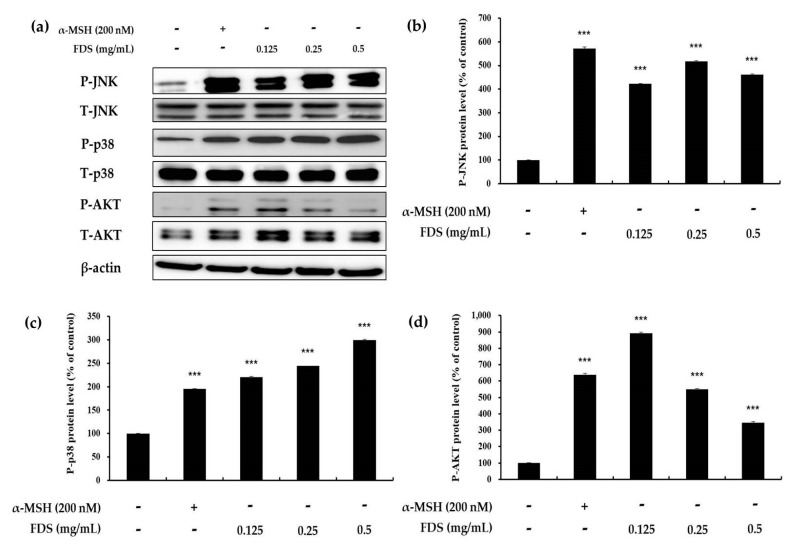
Effects of FDS on phosphorylation of P-JNK, P-p38, and P-AKT. The B16F10 cells were treated with FDS at the indicated concentrations. (**a**) Result of Western blotting and protein levels of (**b**) P-JNK, (**c**) P-p38, and (**d**) P-AKT. The data are presented as mean ± SD of at least three independent experiments. *** *p* < 0.001 vs. untreated cell. JNK: c-Jun N-terminal kinase. AKT: protein kinase B. S: standard deviation. P: phosphorylated. T: total.

Protein kinase B (AKT), p38, and JNK are known to be involved in melanin synthesis. A decrease in the phosphorylation of AKT leads to increased melanin production, whereas an increase in the phosphorylation of p38 and JNK ultimately increases melanin synthesis. Therefore, to analyze the effect of FDS on these signaling pathways, B16F10 murine cells were treated with different concentrations of FDS (0.125 to 0.5 mg/mL). α-MSH was used as a positive control. As shown in Figure 5, FDS enhanced the expression of p38 and JNK, which increases melanogenesis as the concentration of FDS increased. The expression of both phosphorylated p38 and phosphorylated JNK increased with a growing FDS concentration. However, AKT expression did not change in a dose-dependent manner. These results indicated FDS was related to JNK and p38 signaling pathway in enhancing melanogenesis in B16F10 cells.

## 3. Discussion

Skin color is a major issue all around the world because the skin protects all other important organs of the body from various invaders and toxic radiation. Melanin plays a key role by providing skin color and blocking UV radiation from entering the body.

Similarly, hair is another important and major issue around the globe. Hair coloring is directly connected to beauty and attractiveness. Hair whitening is considered undesirable and has now become a major problem with many people suffering from this condition. People use dyes to color their hair, and some natural compounds and anti-hair whitening agents, and, thus, the demand for these coloring compounds is increasing.

To develop a new melanogenic active compound, we used various antibiotics, such as gentamycin and kanamycin (data not shown). Among these antibiotics, FDS was found to be effective. To elucidate its cytotoxicity, we performed the MTT assay on the selected range of concentration of 0.125–0.5 mg/mL, and the results showed that FDS at these concentrations exerted no cytotoxicity, which showed cell viability of almost 100%. Therefore, FDS at this range of concentration was selected for further experiments. Melanocytes can be stimulated by many factors. In this study, we used α-MSH (200 nm) to stimulate the melanogenesis pathway in mouse melanoma cells. To investigate the effect of FDS on cellular tyrosinase activity and cellular melanin content, B16F10 murine melanoma cells were treated with various concentrations of FDS for 72 h. FDS increased both cellular tyrosinase activity and cellular melanin content in a concentration-dependent manner.

In the melanogenesis pathway, tyrosinase, TRP-1, and TRP-2 are the key enzymes. Therefore, induction of these enzymes can cause excess formation of melanin, whereas inhibition of these enzymes is a major strategy to reduce melanin formation in the development of new cosmetics products. The effect of FDS on intercellular tyrosinase activity was investigated in B16F10 murine melanoma cells, and the results showed that FDS increased tyrosinase activity in a dose-dependent manne. Furthermore, the Western blotting result provided clear evidence that FDS induced melanin production by increasing the expression of all melanogenic enzymes without exerting cytotoxicity.

Furthermore, Western blotting analysis was also used to clarify the effect of FDS on the phosphorylation of enzymes of the JNK, p38, and AKT pathways. A recent study showed that decreasing the phosphorylation of AKT increases melanogenesis [14]. Therefore, the phosphorylation of AKT was examined. However, FDS did not change AKT expression in a dose-dependent manner. Previous reports also suggested that an increase in the phosphorylation of p38 and JNK leads to upregulation of melanogenesis. In this study, FDS increased the expression of both phosphorylated p38 and JNK. Taken together, our results provided clear evidence that FDS can be used as an anti-hair whitening agent, as FDS increased melanogenesis and the activity of melanogenesis-related enzymes through the JNK and p38 signaling pathways.

## 4. Materials and Methods

### 4.1. Materials

Fosfomycin disodium salt was purchased from Tokyo Chemical Industry Co., Ltd. (Chuo-ku, Tokyo, Japan). Dimethyl sulfoxide (DMSO), α-MSH, NaOH, MTT, radioimmunoprecipitation assay (RIPA) buffer, and L-DOPA were obtained from Sigma–Aldrich (St. Louis, MO, USA). Dulbecco’s modified Eagle’s medium (DMEM), fetal bovine serum (FBS), penicillin/streptomycin, and trypsin–ethylenediaminetetraacetic acid were purchased from Thermo Fisher Scientific (Waltham, MA, USA). Antibodies against p-p38, p38, p-JNK, JNK, p-ERK, ERK, p-AKT, AKT, and β-actin were procured from Cell Signaling Technology (Danvers, MA, USA). Enhanced chemiluminescence (ECL) kit and 2× Laemmli sample buffer were obtained from Biosesang (Seongnam, Gyeonggi-do, Korea) and Bio-Rad (Hercules, CA, USA), respectively.

### 4.2. Cell Culture

B16F10 murine melanoma cells were supplied by the Korean Cell Line Bank, and supplemented with 10% Fetal bovine serum (FBS) and 1% penicillin-streptomycin. Cells were maintained and incubated in humidified atmosphere with 95% air and 5% CO_2_.

### 4.3. Measurement of Cell Viability

Cell viability was determined using the MTT dye. B16F10 mouse cells at 3 × 10^4^ cells were seeded in 24-well plates and incubated in humidified atmosphere with 95% air, 37 °C, and 5% CO_2_ for 24 h. The cells in each well were then treated with different concentrations of FDS, which was followed by 25 µL of 0.1% MTT and incubated for 2 h. The medium was then carefully removed and replaced with 500 µL of dimethyl sulfoxide (DMSO) to dissolve the formed formazan. The plates were then shaken well on a shaker, and the absorbance was measured at 540 nm. The percentage of cell viability was determined according to a previously described method [14,31].

### 4.4. Measurement of Melanin Content

Melanin content in B16F10 cells was assayed according to a previously described method with a slight modification [14,31]. Cells at 1 × 10^5^ were seeded in 60-mm dishes and incubated for 24 h. After that, the cells were treated with new medium containing 200 nm α-melanocyte-stimulating hormones (α-MSH) and FDS at various concentrations and incubated for 72 h. Next, the medium was carefully removed, and the cells were washed with cold phosphate-buffered saline. Cell pellets were dissolved with 10% dimethyl sulfoxide (DMSO) containing 1N NaOH, and kept in a UV sterilizer at 70 ℃ for 1 h. The experiment was carried out at least three times, and melanin content was measured at 405 nm using an ELISA (Sunrise, TECAN, Mannedorf, Switzerland) reader.

### 4.5. Intracellular Tyrosinase Activity

Intracellular tyrosinase activity assay was performed according to a previously described method with a slight modification [14,31]. α-MSH was used as a positive control. Cells at 1 × 10^5^ were seeded in 60-mm π dishes and incubated for 24 h, and then lysed with phosphate buffer containing 1% Triton X-100. Next, 200 µL radioimmunoprecipitation assay (RIPA) buffer containing a 1% enzyme inhibition cocktail was added to the lysates. The lysates were then clarified by centrifugation for 20 min at 15,000 rpm. After that, 150 µL of protein was carefully collected, placed in a separate tube, and the amount of protein in each lysate was quantified using the Bradford standard assay (BSA). Next, 20 μL of each quantified lysate was mixed with 15 mM of L-DOPA in a 96-well plate and then incubated for 30 min at 300 rpm and 37 °C. The experiment was carried out at least three times, and melanin content was measured at 405 nm using an ELISA (Sunrise, TECAN, Mannedorf, Switzerland) reader.

### 4.6. Western Blotting

B16F10 murine melanoma cells at 1 × 10^5^/well were seeded in 100-mm dishes for 24 h. Various concentrations of FDS (0.125–0.5 mg/mL) were added to the cells, which were then incubated for 72 h. Cell pellets were then collected, and the cells were lysed with radioimmunoprecipitation assay (RIPA) buffer containing a 1% protease inhibition cocktail. The lysates were scrapped. The cell lysates were collected and centrifuged at 15,000 rpm and −10 °C for 20 min. The supernatants were collected from the surface carefully and normalized with the Bradford standard assay (BSA) protein. Next, a 2x Laemmli sample and the supernatants were mixed at the ratio of 1:1 using the BSA table in an e-tube to prepare samples by Western blotting. Each sample contained an equal concentration of 25 µg of proteins. The samples were then heated at 100 °C for 5 min, and 20 µL of each sample was loaded onto a 10% sodium dodecyl sulfate (SDS)-polyacrylamide gel and electrophoresed. Isolated proteins were transferred onto a polyvinylidene difluoride (PVDF) membrane, which was then blocked with 5% non-fate skim milk in Tris Buffered Saline with Tween (TBST) buffer with shaking for 2 h. The membrane was then washed thrice with TBST buffer for 10 min each time. The membrane was then incubated with a rabbit primary antibody diluted to 1:1000 with TBST buffer for overnight, washed with TBST three times, and further incubated for 2 h with secondary anti-rabbit antibody (1:3000) times diluted. Protein bands were visualized with an Enhanced chemiluminescent (ECL) kit. The amount of protein expression was quantified and graphed using the ImageJ program (NIH, Bethesda, MD, USA).

### 4.7. Statistical Analysis

All the data were shown as the mean ± standard deviation of at least three replicates. The results were analyzed using a Student’s *t*-test. Statistical significance was considered at * *p* < 0.05, ** *p* < 0.01, or *** *p* < 0.001.

## 5. Conclusions

**Figure 6:**
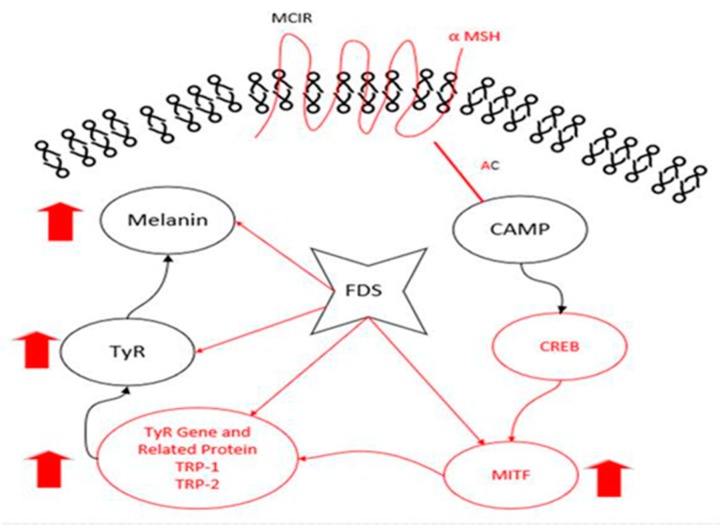
FDS induced melanogenesis by increasing the expression of MITF, TRP-1, TRP-2, and tyrosinase in B16F10 cells. α-MSH: α-melanocyte-stimulating hormone. MC1R: melanocortin 1 receptor. AC: adenylate cyclase. cAMP: cyclic adenosine monophosphate. CREB: cAMP-Responsive Element Binding. MITF: Microphthalmia-Associated Transcription Factor. TYR: tyrosinase. FDS: fosfomycin disodium salt.

This study showed that FDS was a melanogenically active antibiotic that enhanced melanin and tyrosinase content in a dose-dependent manner. It also increased the expression of tyrosinase-related proteins, namely TRP-1, TRP-2, MITF, and tyrosinase, in a concentration-dependent manner (Figure 6). Furthermore, Western blotting results showed that FDS enhanced the production of melanin by upregulating phosphorylated p38 and JNK in a concentration-dependent manner, without causing cytotoxicity in B16F10 cells. Therefore, FDS can be used as an anti-whitening and anti-hypopigmentation agent for hair.
